# Gene encoding γ-carbonic anhydrase is cotranscribed with *argC *and induced in response to stationary phase and high CO_2 _in *Azospirillum brasilense *Sp7

**DOI:** 10.1186/1471-2180-10-184

**Published:** 2010-07-04

**Authors:** Simarjot Kaur, Mukti N Mishra, Anil K Tripathi

**Affiliations:** 1 School of Biotechnology, Faculty of Science, Banaras Hindu University, Varanasi-221005, India

## Abstract

**Background:**

Carbonic anhydrase (CA) is a ubiquitous enzyme catalyzing the reversible hydration of CO_2 _to bicarbonate, a reaction underlying diverse biochemical and physiological processes. Gamma class carbonic anhydrases (γ-CAs) are widespread in prokaryotes but their physiological roles remain elusive. At present, only γ-CA of *Methanosarcina thermophila *(Cam) has been shown to have CA activity. Genome analysis of a rhizobacterium *Azospirillum brasilense*, revealed occurrence of ORFs encoding one β-CA and two γ-CAs.

**Results:**

One of the putative γ-CA encoding genes of *A. brasilense *was cloned and overexpressed in *E. coli*. Electrometric assays for CA activity of the whole cell extracts overexpressing recombinant GCA1 did not show CO_2 _hydration activity. Reverse transcription-PCR analysis indicated that *gca1 *in *A. brasilense *is co-transcribed with its upstream gene annotated as *argC*, which encodes a putative *N*-acetyl-γ-glutamate-phosphate reductase. 5'-RACE also demonstrated that there was no transcription start site between *argC *and *gca1*, and the transcription start site located upstream of *argC *transcribed both the genes (*argC-gca1*). Using transcriptional fusions of *argC*-*gca1 *upstream region with promoterless *lacZ*, we further demonstrated that *gca1 *upstream region did not have any promoter and its transcription occurred from a promoter located in the *argC *upstream region. The transcription of *argC-gca1 *operon was upregulated in stationary phase and at elevated CO_2 _atmosphere.

**Conclusions:**

This study shows lack of CO_2 _hydration activity in a recombinant protein expressed from a gene predicted to encode a γ-carbonic anhydrase in *A. brasilense *although it cross reacts with anti-Cam antibody raised against a well characterized γ-CA. The organization and regulation of this gene along with the putative *argC *gene suggests its involvement in arginine biosynthetic pathway instead of the predicted CO_2 _hydration.

## Background

Carbonic anhydrases (CAs, EC 4.2.1.1) are zinc metalloenzymes which catalyze the reversible hydration of carbon dioxide to bicarbonate (CO_2 _+ H_2_O ↔ HCO_3_^- ^+ H^+^). This simple interconversion of a membrane-permeable gas substrate into a membrane-impermeable ionic product is vital to many important biological functions; such enzymes are thus widely distributed in nature. On the basis of differences in amino acid sequence and structure, carbonic anhydrases are divided into five distinct, evolutionarily unrelated gene families: α, β, γ and the recently discovered δ and ζ [1-4]. The α-CAs are distributed in animals, plants, algae and bacteria. In mammals various α-CA isoforms with different subcellular localization and tissue distribution are implicated in many physiological processes such as carboxylation/decarboxylation reactions, transport of CO_2 _and/or HCO_3_-, pH regulation, ion exchange, calcification, metabolism of urea, glucose and lipids, tumorigenicity, bone resorption and many other physiological and pathological processes [5]. Members of β-CAs are predominant in plants, algae, archaea and bacteria. In photosynthetic organisms β-CAs play an important role in transport and autotrophic fixation of CO_2 _while in prokaryotes β-CAs are involved in wide range of cellular functions including provision of HCO_3_^- ^for carboxylating enzymes which catalyze key steps in biosynthetic pathways for essential metabolites, such as amino acids, nucleotides, fatty acids [6,7].

The γ-CAs are predominant in bacteria and archaea domains. In eukaryotes, they have so far been described only in photosynthetic organisms. While the physiological role of α-CAs in mammals and β-CAs in plants and prokaryotes, have been extensively studied, the role of γ-CAs remain elusive. To date, the only γ-CA that has been extensively characterized is "Cam" from the methanogenic archaeon *Methanosarcina thermophila *[8,9]. In the cyanobacterium *Synechocystis*, the bifunctional CcmM protein localized in carboxysome (structure involved in CO_2 _concentration) shows an N-terminal γ-CA like domain which has been proposed to bind HCO_3_^-^/CO_2 _[10]. However, no carbonic anhydrase activity could be detected for the recombinant CcmM expressed in *E. coli*. Recently, a similar role for binding and transporting bicarbonate has been proposed for γ-CA subunits of plant mitochondrial complex, suggesting that the so-called γ-CAs in photosynthetic eukaryotic organisms do not act as carbonic anhydrases but may have related activity contributing to CO_2 _recycling in photorespiration, or play a role in the carbon transport between mitochondria and chloroplasts to increase the efficiency of photosynthetic CO_2 _fixation [11].

Unraveling of microbial genome sequences has shown that γ-CAs are widespread in prokaryotes, and it is likely that these enzymes play diverse roles in microorganisms. Investigations into the ways in which archaea and bacteria domains use γ-carbonic anhydrase may reveal novel aspects of prokaryotic physiology. We are analyzing the role of carbonic anhydrases in a nonphotosynthetic, Gram-negative, plant growth promoting α-proteobacterium, *Azospirillum brasilense *that lives in close association with the roots of several important crop plants and grasses and stimulates the growth of its host plant by producing phytohormones and siderophores [12]. Earlier, we have cloned the gene encoding β-CA from *A. brasilense*, overexpresed, purified and characterized β-CA. We also showed that the transcription of *bca *gene was down regulated by stationary phase, elevated CO_2 _and acidic pH [13]. The objective of this study was to study the role of a putative gene encoding γ-carbonic anhydrase in *A. brasilense *Sp7.

## Results

### Sequence and phylogenetic analysis of *gca1 *of *A. brasilense*

A search for the presence of ORFs annotated as carbonic anhydrase in the genome of *A. brasilense *Sp245 http://genome.ornl.gov/microbial/abra/ revealed three ORFs out of which two were annotated to encode carbonic anhydrase/acetyltransferase. BLAST results of the amino acid sequences of these two ORFs showed homology with putative γ-CAs. Using the sequence information from *A. brasilense *Sp245 genome, one of the putative γ-CA ORF (*gca1*) of *A. brasilense *Sp7 was PCR amplified, and sequenced. The nucleotide and deduced amino acid sequence of the *A. brasilense *Sp7 *gca1 *and the putative γ-CA of *A. brasilense *Sp245 were 97% and 99% identical, respectively. The *gca1 *ORF consisted of 519 bp, which can translate a polypeptide of 173 amino acids with a predicted molecular mass of 19 kDa. BLASTP analysis of the deduced amino acid sequence of *A. brasilense *Gca1 revealed 27% identity with Cam, a γ-CA from *M. thermophila*. In addition to its homology with putative γ-CAs, Gca1 also showed significant homology to proteins annotated as acetyltransferase/isoleucine patch superfamily with no predicted function (unknown proteins).

As inferred from X-ray crystallographic studies of Cam, the active-site zinc is coordinated by three histidine residues [9]. The alignment of Gca1 with the Cam sequence showed that the essential histidines (His-81, His-117 and His-122) required for ligating the active site Zn are absolutely conserved in Gca1. Further analysis revealed that three other residues (Arg-59, Asp-61 and Gln-75) present in all γ-class CA sequences and reported to be involved in biochemical activity of Cam of *M. thermophila*, are also conserved in Gca1 (Additional file 1 Figure S1). Two glutamate residues, Glu-62 and Glu-84 of Cam, whose role has been shown in CO_2 _hydration and proton transfer, respectively, are conserved in cyanobacterial CcmM sequence but neither in Gca1 nor in other γ-CA homologues such as *Pseudomonas putida *(PhaM) and *E. coli *(CaiE) which share 36%, and 32% identity, respectively, with Gca1, suggesting that alternative residues might serve these roles.

**Figure 1:**
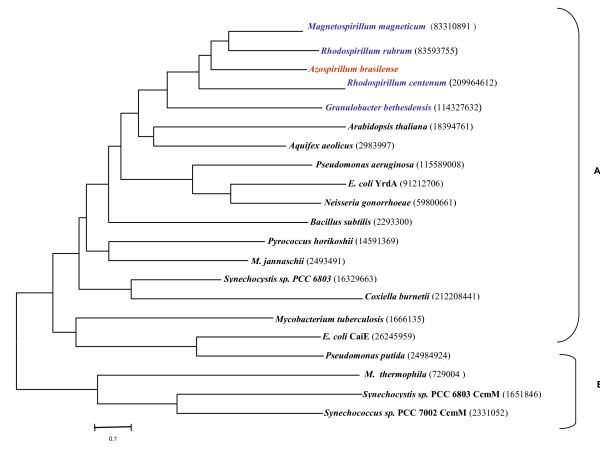
**Phylogenetic tree based on neighbor-joining analysis of amino acid sequences of γ-CA from *A. brasilense *and other organisms**. Putative γ -class carbonic anhydrase sequences were aligned using Clustal W and analyzed with the MEGA version 4.0 [28]. The 2 phylogenetic clades are indicated by bars on the right. The GenBank accession numbers for the sequences used are indicated in parentheses.

To examine the phylogenetic relationship of *A. brasilense *Gca1 with other known orthologs, the amino acid sequences of different γ-CAs from eukaryotic photosynthetic organisms, cyanobacteria, bacteria and archaea were used to generate multiple sequence alignment and a phylogenetic tree (Figure 1). The deduced γ-CA amino acid sequences clustered in two clades; the larger Clade A consisted of sequences from all three domains of life. The catalytically important residues of Cam, Glu-62 and Glu-84 were missing in these sequences and information regarding CA activity of protein encoded by any of these sequences is lacking. Clade B consisted of well documented Cam protein from *M. thermophila *and cyanobacterial CcmM proteins. Although Cam has been shown to biochemically function as CA and its physiological role during acetotrophic growth has been suggested, no CA activity for CcmM, the closest relative of this CA in the phylogeny has been reported.

Phylogenetic analysis suggests that γ-class is largely populated with homologs of a subclass that lack proton shuttle residues essential for Cam, and the deduced Gca1 sequence of *A. brasilense *falls in this subclass along with orthologs from closely related members of α- proteobacteria, viz. *Magnetospirillum magneticum, Rhodospirillum rubrum, Rhodospirillum centenum *and *Granulibacter bethesdensis*.

### Analysis of *gca1 *gene transcript in minimal and rich medium

**Figure 2:**
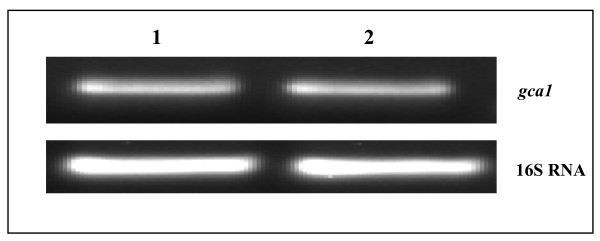
**Agarose-gel showing amplified products obtained by reverse transcriptase-polymerase chain reaction (RT-PCR) with total RNA isolated from *Azospirillum brasilense *Sp7 grown in minimal (lane 1) and rich medium (lane 2)**. Lower strip is showing the amplification of 16 S RNA from the same amount of RNA sample as a control.

Before extending the study on functional analysis of *gca1 *in *A. brasilense*, the expression of *gca1 *gene in *A. brasilense *cells was examined. Cell extracts of *A. brasilense *showed very low level of carbonic anhydrase activity of 0.3 ± 0.1 U/mg. Since *A. brasilense *genome also encodes a functional β-CA [13], it was not clear if the observed CA activity was due to β-CA or also due to γ-CA. To determine whether *gca1 *is expressed in *A. brasilense *under ambient conditions, RT-PCR with RNA samples isolated from the mid-log phase cultures grown in minimal (MMAB) or rich (LB) medium was performed. The ~500 bp *gca1 *transcripts was produced from both the RNA samples (Figure 2) which was confirmed by sequencing the cDNA amplicons. These results indicated that *A. brasilense gca1 *is constitutively expressed in cells grown in minimal or rich medium under ambient atmospheric conditions.

### Characterization of protein encoded by *gca1*

**Figure 3:**
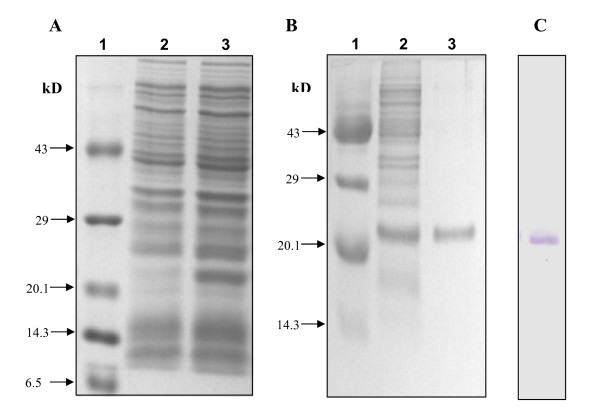
**Heterologous overexpression, purification and western blot analysis of recombinant Gca1 of *A. brasilense ***(A) **SDS-PAGE gel electrophoresis (15%) of uninduced (lane 2) and induced (lane 3) cell lysates of transformants harboring pSK7**. The Gca1 protein overproduced in *E. coli *pSK7 is encircled. Low range molecular weight marker, Bangalore Genei (lane 1). **(B)**. Purification of recombinant Gca1 of *A. brasilense *under denaturing conditions SDS-PAGE gel (15%) showing induced crude extract of transformant harboring pSK7 (Lane 2); Ni-NTA purified His.Tag Gca1 (Lane 3); Low range molecular weight marker, Bangalore Genei (Lane 1). **(C) **Western blot analysis showing cross-reactivity of purified recombinant Gca1 with antisera raised against CAM.

To examine whether *gca1 *gene encoded a functionally active protein, the *gca1 *ORF was amplified from the *A. brasilense *Sp7 genomic DNA and directionally cloned into the pET15b to construct an over-expression plasmid, pSK7 which, after confirmation by sequencing, was used for expression in *E. coli *and purification of the recombinant protein. SDS-PAGE analysis of extracts from uninduced versus induced cultures showed the presence of a protein of the expected size in the induced cells (Figure 3A). The size of the recombinant Gca1 (ca. 21 kDa) was larger than the predicted polypeptide size (19 kDa) due to the additional vector-encoded His-tag at the N-terminus of the protein. The recombinant protein was purified to homogeneity under denaturing conditions (Figure 3B). A clear band of purified protein in the position corresponding to the overexpressed protein in the crude lysate was visualized on the gel (Figure 3B). This band cross-reacted with anti-Cam antiserum (Figure 3C). The recognition of recombinant Gca1 with heterologous antibody indicates significant similarity between Gca1 and Cam.

No CA activity could be detected in crude cell extracts of *E. coli *overexpressing recombinant Gca1 while under the same CA activity assay conditions, α-bovine CAII showed specific CA activity of about 1024 WAU/mg, respectively. These results indicate that the supernatant fractions containing soluble recombinant Gca1 lacked detectable CO_2 _hydration activity.

### Construction of *gca1 *knockout (Δ*gca1*) mutant

In order to gain an insight into the possible physiological role of Gca1 in *A. brasilense*, attempt was made to construct a *Δgca1 *of *A. brasilense *Sp7 by inserting kanamycin resistance gene cassette into the coding region of *gca1 *but in spite of repeated attempts no *gca1 *mutant could be isolated. Since deletion of CA gene generally results in high CO_2 _requiring (HCR) phenotype [14], attempts were also made to isolate the desired mutants at 3% CO_2 _concentration (the highest CO_2 _concentration at which *A. brasilense *Sp7 is able to grow). The inability to obtain γ-CA knock-out mutant under aerobic atmosphere as well as under the atmosphere containing 3% CO_2 _probably reflects that this putative γ-CA might be essential for the survival and growth of *A. brasilense *in the atmosphere containing ambient to 3% levels of CO_2_. Since bicarbonate is a substrate for carboxylating enzymes central to many metabolic processes [6], attempts were also made to restore *Δgca1 *by supplementing the minimal medium with some metabolic intermediates (as mentioned in Methods). Unfortunately, none of these supplements rescued *Δgca1 *of *A. brasilense *suggesting that the putative Gca1 protein might have physiological implications other than hydration of CO_2_.

### Bioinformatic analysis of *gca1 *organization: Prediction of *argC-gca1 *operon in *A. brasilense*

**Figure 4:**
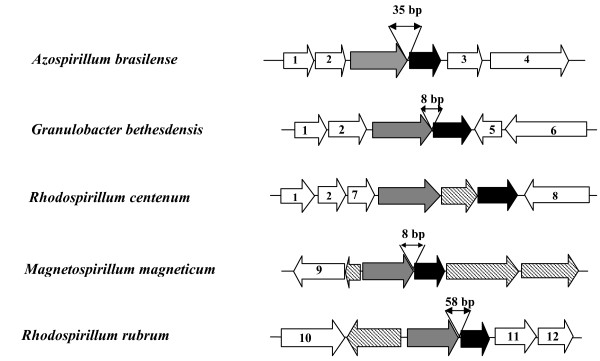
**Schematic representation of the genomic organization of gene predicted to encode γ-CA in *Azospirillum brasilense *and other closely related α-proteobacteria sharing highest similarity for γ-CA sequences**. Arrows indicate the positions and orientations of the potential ORFs predicted to encode γ-CA (black), N-acetyl-gamma-glutamyl phosphate reductase (gray), hypothetical proteins (lined) and other known proteins (white). 1. 50 S ribosomal protein; 2. 30 S ribosomal protein; 3. OmpA/MotB domain protein precursor; 4. Poly(3-hydroxyalkanoate) synthase; 5. phosphoribosyl AMP cyclohydrolase; 6. cystathionine beta lyase; 7. Acetyltransferase (GNAT family); 8. poly-beta hydroxybutyrate transferase; 9. Arylsulphatase regulator; 10. Aminotransferase; 11. ABC transporter component; 12. Binding protein dependent transport systems inner membrane component.

While analyzing the organization of *gca1 *chromosomal region of *A. brasilense *using genome database and NCBI database BLAST resources, a putative gene (annotated as *argC*) was identified that was located upstream of *gca1 *ORF in the same transcriptional orientation with an intergenic distance of 35 nucleotides (Figure 4). The *argC *gene product (351 amino acids) of *A. brasilense *shared high similarity with the ArgC protein of *R. centenum, M. magneticum *and *R. rubrum*. The *N*-acetyl-gamma-glutamate-phosphate reductase (EC 1.2.1.38) encoded by *argC *is involved in the arginine biosynthesis in prokaryotes [15]. The arginine biosynthetic pathway proceeds via N-acetylation of L-glutamate by N-acetylglutamate synthase (ArgA) yielding N-acetylglutamate which is converted into N-acetylglutamyl-phosphate by N-acetylglutamate 5-phosphotransferase encoded by *argB*. N-acetylglutamyl-phosphate is subsequently reduced to N-acetylglutamic semialdehyde by N-acetylglutamyl-phosphate reductase, encoded by the *argC *gene. Thus the ArgC protein catalyses the third step in the pathway of biosynthesis of arginine from glutamate [15].

Several studies have shown that short intergenic distance between ORFs and phylogenetically conserved gene order are important generalized predictor of operon structure [16]. Thus, conservation of this adjacent, co-directional gene-pair might link apparently unrelated *argC *and *gca1 *genes in a co-transcriptional relationship. In order to test this possibility, the chromosomal neighbourhoods of *gca1 *orthologs in sequenced bacterial genomes of the members of phylogenetic tree (Figure 1) including both distant and close relatives of *A. brasilense *were analyzed. Interestingly, this gene order was found to be fairly well conserved in some of the sequenced members of *Rhodobacteriaceae *such as *M. magneticum, R. rubrum *and *R. centenum *(Figure 4). A similar syntenic organization was also observed in a member of *Acetobacteriaceae *(*Granulibacter bethesdensis*), but not in other bacterial genomes in which *gca1 *homologs are found. Examination of the intergenic distance between *argC *and γ-CA encoding genes revealed a distance of only 8 nt in *M. magneticum *and *G. bethesdensis*, 35 nt in *A. brasilense *and 58 nt in *R. rubrum *whereas in *R. centenum *a gene encoding a protein of unknown function present between these two genes. Thus, a conserved gene order of *argC-gca1 *and relatively short intergenic distance in *A. brasilense *and phylogenetically close members suggested that these two adjacent codirectional genes might comprise a bicistronic operon and also the possibility of functional and/or regulatory relationship between the two genes. The synteny with regard to the two other ORFs encoding 30 S and 50 S ribosomal subunit proteins, respectively, located upstream of the *argC *gene was observed in *A. brasilense *as well as in *G. bethesdensis *and *R. centenum *but not in other closely related bacteria.

### Confirmation of the transcriptional linkage of the *argC-gca1 *ORFs

**Figure 5:**
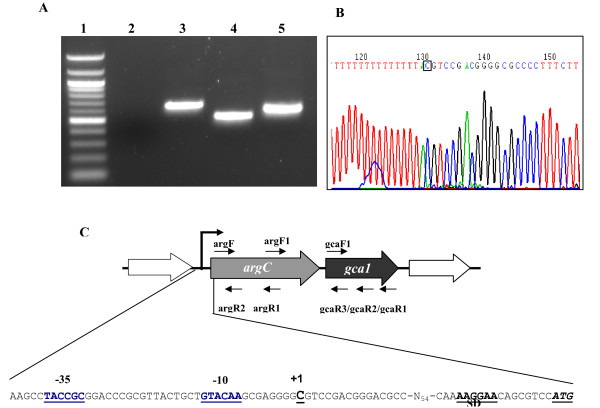
**Determination of *argC*/*gca1 *transcription unit and transcription start site of argC/gca1 transcript. A**. Agarose gel showing amplified products obtained by reverse transcriptasepolymerase chain reaction (RT-PCR) with total RNA isolated from *Azospirillum brasilense *Sp7 using argF/argR1 (Lane 3), gcaF1/gcaR1 (Lane 4) and argF1/gcaR3 (Lane 5) primer sets. Lane 1 and 2 shows the bands of 100 bp DNA ladder (NEB) and control without reverse transcriptase, respectively; **B**. Determination of *argC*/*gca1 *transcription start site by 5' RACE experiment. The electropherogram is representative of results from sequencing of several distinct clones obtained after 5'RACE. The first base (C) downstream of the dT tail-A corresponds to the first nucleotide transcribed or TSS; **C**. Schematic representation of the *argC-gca1 *chromosomal region of *A. brasilense*. Large arrows represent the ORFs, and their orientation shows the transcriptional direction. Small arrows indicate the location of primers used for RT-PCR and 5'RACE experiment. The nucleotides representing TSS (+1), putative -35 and -10 boxes, and SD are underlined. Start codon (ATG) of *argC *is italicized.

To determine if *argC *and *gca1 *genes are part of a single operon and transcribed as a single mRNA, reverse transcription-PCR (RT-PCR) experiments were performed using total RNA isolated from *A. brasilense *cultures using three different primer sets, (Table 1 and Figure 5C) gcaF1/gcaR1 to amplify *gca1 *ORF (519 bp), argF/argR1 for 687 bp portion of *argC *ORF and argF1/gcaR3 to amplify the transcript (625 bp) encompassing both *argC *and *gca1 *ORFs. Analysis of RT-PCR amplified product revealed that argF1/gcaR3 primer set produced a fragment of expected size (ca. 600) indicating that there was a single mRNA for these two genes. Amplicons of expected size, ca 700 bp and ca 500 bp, were also obtained with *argC *and *gca1-*specific primer sets, respectively (Figure 5A). RT-PCR analysis confirmed that these genes are, in fact, co-transcribed which suggests a new functional linkage between the two genes that may have interesting implications for *A. brasilense *physiology.

**Table 1:** Primers used in this study (restriction sites are shown by underlined sequences)

Primers	Sequence (in 5' to 3' direction)
gcaF	**GGAATTC****CAT **ATGTCCGGCCTGATATTGCCC
gcaR	**CGGGATCC **TTAGCCTTCTCTGTAGATTTGAG
gcAF	**AAACTGCAG **ATACGCCACCTGGTACGGGCATG
gcAR	**GAAGATCT**GATGAAGCAGCCGCCCTCCAGC
gcBF	**GAAGATCT **GGACGGTGCCTACGTCGAGTCG
gcBR	**GGAATTC **GAAGTTCGTGCTGGCGGCCTC
gcaPrF	**CGGGGTACC **AGCAGCAGAATCTCTTCACC
gcaPrR	**AAAAGGCCT **GTCACGGGAACAGCGGAG
argPrF	**CGGGGTACC**GAAGTGGTCGCCCCGAAG
argPrR	**AAAAGGCCT**GACGCACGGGGATGGGC
gcaF1	ATGTCCGGCCTGATATTGC
gcaR1	TTAGCCTTCTCTGTAGATTTG
gcaR2	CCATGTGACCGATCGACAC
gcaR3	CACCGATTCGGATCTCGTTCAC
argF	ATGGCCAACAGCACTTCCC
argF1	GTGACGGTCAGCTTCACG
argR1	CATGCGGACGTAGATCGTC
argR2	CTCGATCATCTCATCCATCAGCAG

### Determination of transcription start site of *argC-gca1 *transcript

Co-transcription of *argC-gca1*, confirmed by RT-PCR, prompted us to determine the transcription start site (TSS) and promoter elements involved in the regulation of this operon. We were also interested to examine if *gca1 *has its own TSS which could be used to regulate transcription of only *gca1 *from a promoter located upstream of *gca1 *somewhere in *argC *ORF. For this purpose, we performed 5'RACE experiment using RNA sample isolated from *A. brasilense *Sp7. In the first step of 5'RACE experiment, we used gcaR1 for cDNA synthesis as this primer could drive the synthesis of cDNAs from both types of transcripts (from *argC-gca1 *and *gca1*), if present. In the later reactions, the respective nested primers were used (as described in materials and methods) to amplify regions upstream of *argC *and *gca1*. Amplicons obtained in both cases, with *gca1 *and *argC *specific nested primers, showed a single transcription start from a C residue located at position -94 relative to the predicted translational start site of *argC *(Figure 5B and 5C) indicating the presence of only one TSS for this predicted operon located upstream of *argC *ORF. Analysis of the region upstream the identified TSS for corresponding promoter elements (sequences at -35 and -10 regions) indicated the presence of CTACCG at -35 and GTACAA at -10 of TSS with a spacing of 17 nt. Eight base pairs upstream from the ATG initiation codon, a consensus AAGGAA Shine-Dalgarno sequence for ribosome binding was found (Figure 5C).

### Inducibility of *argC-gca1 *operon in response to stationary phase and high CO_2_

After the confirmation of co-transcription by RT-PCR and determination of transcription start site by 5'RACE experiment which suggested the transcription of *argC *and *gca1 *genes from a promoter located upstream of *argC *ORF, we examined the regulation of *argC-gca1 *operon in response to different conditions. For this purpose, - 455 to + 79 of TSS of *argC-gca1 *was inserted upstream of the promoterless *lacZ *reporter in pRKK200 to make transcriptional fusion (pSK8), and β-galactosidase assay was performed with cells of *A. brasilense *harboring pSK8 and grown in MMAB in different conditions.

**Figure 6:**
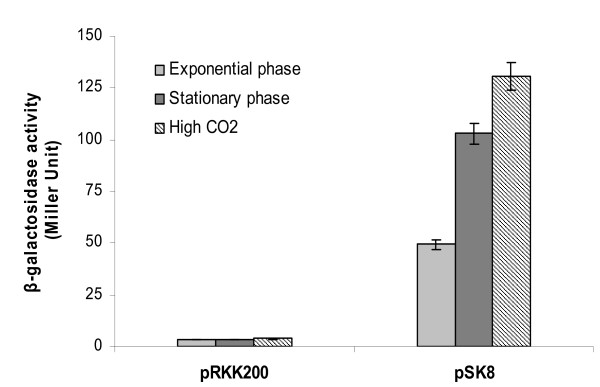
**27 Effect of growth phase and CO_2 _concentration on *argC*-*gca1 *promoter activity β-galactosidase assay was performed with *A. brasilense *Sp7 cells harbouring either pRKK200 (empty vector) or pSK8 and grown up to either exponential or stationary phase at ambient air, and exponential growing cells at high CO_2 _concentration**. The assay was performed on two different occasions. The error bars indicate standard deviation from the three replicates.

Comparison of β-galactosidase activity in the cells taken from exponential and stationary phase cultures **(**Figure 6) showed that P_argC _activity was significantly up-regulated (more than 2 fold) during stationary phase than in the exponential phase of growth. Similarly, β-galactosidase activity measured in exponentially growing cells of *A. brasilense *harboring pSK8 under 3% CO_2 _enriched atmosphere was ~3 fold higher than the cells grown in ambient atmosphere (Figure 6). These data suggested that the P_argC _is constitutively but weakly expressed in exponentially growing cells under optimal growth conditions but significantly induced in response to high CO_2 _or stationary phase.

In order to further confirm whether *gca1 *has its own promoter, an additional construct (pSK9) was made by inserting -501 to - 11 of predicted translational start of *gca1 *in the same vector (pRKK200). No β-galactosidase activity could be detected with cells of *A. brasilense *strains harboring pSK9 under any of the above conditions (data not shown) indicating that there is no promoter upstream of *gca1*. This result further confirmed the previously noted single TSS by 5'RACE experiment for *argC-gca1 *operon and no independent transcription start site for *gca1*. Thus the results obtained from 5'RACE experiment and promoter analysis is in agreement with the notion that transcription of *argC*-*gca1 *operon is regulated by a single promoter located upstream of *argC*.

As *argC *is involved in arginine biosynthesis in prokaryotes, and arginine biosynthetic genes are normally induced in response to arginine limitation as might be the case in stationary phase when arginine becomes limiting [17]. To ascertain if the induction of P_argC _in stationary phase is a consequence of arginine limitation, promoter activity assay was performed with the cells harbouring pSK8 taken from exponential phase and stationary phase cultures grown in minimal media supplemented with L-arginine (0.1, 0.5, 1mM). No difference was found in the β-galactosidase activity in cultures lacking/supplemented with exogenous arginine (data not shown). As supplementation with exogenous arginine did not affect the activity of P_argC _in either exponential or stationary phase, it is likely that regulation of expression of *argC-gca1 *operon is arginine independent.

## Discussion

Availability of bacterial genome sequences has opened a new range of possibilities to elucidate the functions of these sequences, thus providing biochemical, physiological, evolutionary, and ecological meaning to the nucleotide sequence data. Release of partial genome sequence of *A. brasilense *has allowed the characterization of different genes that might be involved in the physiology of this plant growth promoting bacterium. *A. brasilense *genome revealed the presence of one β-CA and two putative γ-CA encoding genes. Recently, we have shown that β-CA gene in *A. brasilense *encoded a functionally active protein, and its expression was regulated by growth phase, CO_2 _concentration and pH [13]. In this work, one of the putative ORFs whose amino acid sequence shared significant identity with other members of the γ-CA family was characterized.

The cell-free extracts having overexpressed recombinant Gca1 protein did not show CA activity under the conditions tested. Similar lack of detectable CA activity as found in case of recombinant Gca1 protein was also observed in recombinant γ-CA of *Arabidopsis *[18], two cyanobacterial CcmM orthologs [10], *E. coli *proteins YrdA, CaiE, and PaaY [19], γ-CA-like proteins from *C. glutamicum *[6] and *C. reinhardii *[20]. It is interesting to note that since the discovery of CA activity in Cam in 1994, all reported tests for CA activity in Cam homologs have proven negative although structural modelling and sequence analyses showed homology with the overall fold of Cam and conservation of the residues essential for metal binding and catalysis, except Glu-62 and Glu-84. Also, antibodies directed against Cam specifically recognized Gca1 (Figure 3C) and mitochondrial γ-CAs [18].

As no Δ*gca1 *mutant could be isolated under the tested conditions, the functional role of Gca1 was analyzed by examining its neighboring genes. Conservation of the gene order in prokaryotes has been considered as one of the important predictors of gene function that helps in speculating the function of a gene based on its neighborhood or gene organization [16]. The inspection of the genome sequences of other bacteria revealed that the Gca1 homologues found in bacteria phylogenetically close to *A. brasilense *had a striking synteny for *gca *locus. On the basis of short intergenic distance and phylogenetically conserved organization of *argC-gca1*, an operon-like organization of the two genes, *argC *and *gca1 *in *A. brasilense *was predicted. RT-PCR analysis revealed a transcript encompassing *argC *and *gca1 *genes confirming that *argC*-*gca1 *genes were co-transcribed in *A. brasilense*. In addition, 5'RACE experiment confirmed a single transcription start site located upstream of *argC*, and a lack of independent TSS for *gca1*. One of the major advantages of operon prediction in relatively less investigated organisms is that in many cases we may be able to link hypothetical genes to more-well-characterized loci and thus gain some insight into the possible function and regulation of the uncharacterized gene(s).

As γ-CAs of plant mitochondrial complex have recently been proposed to be involved in binding/transporting CO_2_/bicarbonate [11], it is intriguing to note that the putative γ-CAs of α- and γ-proteobacteria (considered as mitochondrial ancestors) may also be inactive with respect to CA activity and only bind CO_2 _and/or bicarbonate in the context of different physiological processes. In the present case, on the basis of the induction of *argC-gca1 *promoter activity in response to high CO_2_, and lack of detectable CA activity of Gca1, it can be speculated that Gca1, like mitochondrial γ-CA, might also be involved in binding of CO_2_/HCO_3_^- ^to provide the substrates to different metabolic enzymes, and may not act as carbonic anhydrase.

The amino acid sequence of γ-CAs also showed significant similarity with proteins belonging to hexapeptide repeat family composed mainly of acetyl transferases [21-23] and since the biosynthesis of arginine from glutamate proceeds through several N-acetylated intermediates [15], it is possible that Gca1 might be involved in the acetylation of some intermediate/s in the arginine biosynthetic pathway.

Promoter activity data also indicate that the regulation of *argC-gca1 *promoter is not affected by exogenous arginine. The lack of repression of the *A. brasilense argC*-*gca1 *genes by arginine is consistent with the data reported on the activities of arginine biosynthetic enzymes in various bacteria and cyanobacteria that exhibit a cyclic pathway of ornithine synthesis, where the regulatory mechanism appears to rely mostly on feedback inhibition by arginine of the second enzyme, N-acetylglutamate phosphotransferase [15]. Under nutrient-limiting conditions during stationary phase, arginine is an important metabolite as it can act both as a carbon and nitrogen source. Arginine is also a precursor for the synthesis of polyamines, putrescine and spermidine, which may reduce oxidative damage to proteins and DNA. Since in *E. coli*, arginine constitutes 11% of the cell's nitrogen in stationary phase, biosynthesis of this amino acid is thought to be important under sub-optimal conditions [17]. This is the first report showing the role of CO_2 _in the regulation of *arg*C expression in any bacteria. Although the precise role of *arg*C in arginine biosynthesis in *A. brasilense *is not yet established, it is likely that the high metabolic CO_2 _generated during stationary phase up-regulates arginine biosynthetic genes, including *argC-gca1 *operon alleviating arginine limitation in the nutrient starved stationary phase cells. The induction of *argC-gca1 *operon during stationary phase and at high CO_2 _observed in this study suggests a possible regulatory link between arginine metabolism and another not yet characterized carbon dioxide-dependent process in which Gca1 like protein might have a role to play.

## Conclusion

This study shows lack of CO_2 _hydration activity in the recombinant γ-CA-like protein from *A. brasilense*. The unique operonic organization of *gca*1 and *argC*, observed in *A. brasilense *is syntenous with some of its closely related α-proteobacteria, viz. *Magnetospirillum, Rhodospirillum, Granulibacter *etc. This suggests that the γ-CA-like gene cotranscribed with *arg*C gene in *A. brasilense*, instead of being involved in CO_2 _hydration, may have a role in arginine biosynthesis.

## Methods

### Bacterial strains, culture conditions, plasmids and chemicals

Strains and plasmids used in this study are listed in Table 2. *A. brasilense *Sp7 was grown in minimal medium (MMAB) containing malate (37 mM) and NH_4_Cl (10 mM) as sole source of carbon and nitrogen, respectively [24] or on Luria-Agar at 30°C. *E. coli *strains like DH5α (Gibco-BRL), S.17.1 were grown in Luria-Bertani (LB) medium and BL21λ (DE3) pLysS (Novagen) in Terrific broth (TB) medium at 37°C in the presence of appropriate antibiotics where required. *E. coli *DH5α was used as plasmid host and BL21λ (DE3) pLysS was used as expression system. Plasmid pET15b (Novagen) and pRKK200 [25] were used for expression and for construction of promoter: *lacZ *fusions, respectively. All chemicals used for growing bacteria were from Hi-media (India), chemicals used in enzymatic assays were purchased from Sigma (USA) and enzymes used for DNA modification and cloning were from New England Biolabs (UK). Plasmid isolation kits and gel elution or purification kits were purchased from Qiagen (USA) and Promega (USA), respectively.

**Table 2:** Bacterial strains and plasmids used

Strains or plasmids	Relevant properties	Reference or Source
**Bacterial Strains**		
*E. coli *DH5α	*Δ lacU*169 *hsdR*17 *recA1 endA*1 *gyrA*96 *thiL relA1*	Gibco/BRL
*E. coli *Bl21 λ (DE3) pLysS	*omp*T *hsd*S(r _B_^- ^m_B_^-^) dcm+ Tet^r ^endA gal λ (DE3)	Novagen
*A. brasilense *Sp7	Wild-type strain	[12]
**Plasmids**		
pET15b	Expression vector, Amp^r^	Novagen
pRKK200	Km^r^, Sp^r^, *lacZ*-fusion reporter vector	[25]
pSK7	*gca1 *ORF from *A. brasilense *Sp7 cloned in *Nde*I/*BamH*I site of pET15b	This work
pSJ3	Amplicon A and B cloned in pSUP202 plasmid	This work
pSJ4	Km^r ^gene cassette cloned in BglII site of pSJ1.	This work
pSK8	*A. brasilense argC *promoter region cloned in *Kpn*I/*Stu*I site of pRKK200	This work
pSK9	*A. brasilense gca1 *promoter region cloned in *Kpn*I/*Stu*I site of pRKK200	This work

### Construction of γ -CA expression plasmid

Over-expression construct for heterologous expression of *A. brasilense gca1 *was constructed by cloning (in-frame) the PCR-amplified *gca1 *gene of *A. brasilense *into the expression vector pET15b (Novagen), digested with *Nde*I/*BamH*I. The complete coding region of *A. brasilense gca1 *gene was amplified by PCR using primers gca1F/gca1R (Table 1). The amplicon was digested with NdeI/BamHI, PCR-purified and ligated with the similarly digested expression vector pET15b (Novagen) to generate the plasmid pSK7. *E. coli *DH5α was then transformed with the ligation mix and the transformants were selected on Luria agar with ampicillin (100 μg/ml). After verification of the clones by restriction digestion and sequencing, *E. coli *BL21(DE3) pLysS competent cells were transformed with the plasmid pSK7, and transformants were selected on Luria agar with ampicillin (100 μg/ml) or ampicillin(100 μg/ml)/chloramphenicol (25 μg/ml) respectively.

### Expression, purification and western blot analysis of recombinant Gca1

For expression of recombinant protein, the *E. coli *BL21 (DE3) pLysS cells harboring pSK7 were cultured overnight in Terrific-Broth medium containing appropriate antibiotics at 37°C, diluted with 1:100 fresh medium containing antibiotics and incubated at 37°C with shaking at 150 rpm. When OD_600 _reached a value of about 0.6, the expression of His.tag-Gca1 was induced by adding 1 mM IPTG in the presence of 500 μM ZnSO_4 _for an additional 6 h at 28°C. The cells were harvested by centrifugation and resuspended in lysis buffer (25 mM Tris-SO_4_, pH 8.0, 300 mM NaCl, 1 mM PMSF, 10 mM β-ME, 100 μm ZnSO_4_, 0.1% Triton X-100), lysed with lysozyme (1 mg/ml) followed by sonication at 4°C with six 10 s bursts and 10 s cooling period between each burst. Following centrifugation (10,000 × *g *for 10 min at 4°C), supernatant fractions were run on 15% SDS-PAGE, and stained with Coomassie brilliant blue R-250 (CBB) to determine the profile of recombinant Gca1 expression. The recombinant protein was purified under denaturing conditions using Ni-NTA resin according to manufacturer's instructions (Qiagen, USA). Immunoblots with purified recombinant Gca1 were performed on PVDF membrane (Immobilon, Millipore) (Bio-Rad, USA) using anti-Cam [8] and goat anti-rabbit IgG- alkaline phosphatase conjugate antibodies. The antibody-antigen complex was detected with 5-bromo-4-chloro-3-indolylphosphate and 4-nitroblue tetrazolium chloride.

### Assay for carbonic anhydrase

CA activity in cell extracts was assayed using a modified electrometric method [26]. The assays were performed at 0 to 4°C by adding varying amounts of cell extract (10-100 μl) to 3.0 ml Tris-SO_4 _buffer, pH 8.3, and the reaction was initiated by adding 2.0 ml ice-cold CO_2_-saturated water. The enzyme activity was determined by monitoring the time required for the pH of the assay solution to change from pH 8.3 to 6.3. The pH change resulting from CO_2 _hydration was measured using a Beetrode microelectrode and Dri-Ref system (World Precision Instruments) connected to the pH meter. An α-type bovine CAII (Sigma) was used as a positive control. One Wilbur-Anderson unit (WAU) of activity is defined as (*T*_0 _- *T*)/*T*, where *T*_0 _(uncatalyzed reaction) and *T *(catalyzed reaction) are recorded as the time required for the pH to drop from 8.3 to 6.3 in a buffer control and cell extract, respectively. Protein concentration was determined using the Folin's-Lowry assay using BSA as standard. Specific activity was expressed as WAU/mg of protein.

### Construction of *gca1 *knockout mutant in *A. brasilense *Sp7

Attempt was made to produce *gca1 *knockout mutant (or Δ*gca1 *mutant) of *A. brasilense *Sp7 by replacing the chromosomal wild copy with the mutated copy that was inactivated by inserting kanamycin resistance cassette and located on a suicide plasmid. Primers were designed to amplify *gca1 *gene along with its flanking region in two parts, amplicons A and B. The amplicon A (amplified with primers gcAF/gcAR, Table 1) was of 1050 bp, which included half of the 5' region of *gca*1 with its upstream flanking region. The amplicon B (amplified with primers gcBF/gcBR, Table 1) was of 1453 bp, which has half of the 3'gene with its downstream flanking region. The primers were designed so as to generate restriction sites for *Pst*I at 5' and *Bgl*II at 3' end of the amplicon A, and restriction sites for *Bgl*II at 5' and *EcoR*I at 3' end of the amplicon B. The purified PCR products were digested with the respective enzymes and ligated with the *Pst*I-*EcoR*I digested pSUP202 generating pSJ3. Plasmid pUC4K was digested with *BamH*I and the Km^r ^gene cassette of 1300 bp was eluted and cloned at the *Bgl*II site of pSJ3 to generate final construct designated as '*gca*1 disruption plasmid' or pSJ4 in which the Km^r ^gene cassette had disrupted the *gca1 *ORF. *E. coli *S.17-1 was then transformed with the disruption plasmid, pSJ4 (Table 2) and used as donor in a biparental mating experiment wherein *A. brasilense *Sp7 was used as recipient. The exconjugants were selected on MMAB plates supplemented with Km (40 μg/ml). Several metabolites were used to complement the lack of *gca1 *gene to support the growth of the *gca1 *knockout mutant in 0.033% CO_2 _(air) or in 3% CO_2 _atmosphere. The MMAB was enriched with following combination of nutritional supplements: adenine (20 mg/l), uracil (20 mg/l), L-arginine (20 mg/l), bicarbonate (2 g/l) and a fatty acid mixture containing myristic, stearic and palmitic acids (30 mg/l each) and Tween 80 (10 g/l) as surfactant. Adenine, uracil, L-arginine and bicarbonate were added from filter-sterilized concentrated stock solutions [14]. The fatty acid mixture was added from a 100-fold-concentrated stock solution prepared under sterile conditions. Plates were incubated at 30°C for 7-15 days either under a normal air atmosphere or in a CO_2 _incubator (Thermo-Scientific) with an atmosphere consisting of 3% CO_2_.

### RNA extraction and RT-PCR

Total RNA was extracted from *A. brasilense *cells taken from cultures grown up to late-log phase (2.5 to 2.8 OD_600nm_) using TRIzol reagent (Invitrogen, USA). Isolated sample was treated with 0.05 U RNase free DNAse I (NEB, UK) per μg of RNA for 30 min at 37°C and purified by phenol extraction followed by ethanol precipitation. RT-PCR was carried out with 1-1.5 μg of RNA using one-step RT-PCR kit (QIAGEN, Germany) according to the manufacturer's instructions. The cycling condition used were 50°C for 30 min; 95°C for 15 min; and 30 cycles of 95° for 30 sec, 52-58°C (according to the primer used in reaction) for 30 sec and 72°C for 1 min, followed by incubation at 72°C for 10 min. Negative controls were made with PCR to check for DNA contamination.

### 5' RACE Experiment

The transcription start site (TSS) for *argC *and *gca1 *genes were determined by 5'RACE experiment using the 3'/5'RACE kit, 2^nd ^Generation (Roche, Germany) according to manufacturer's instructions. Briefly, total RNA was isolated from the cells taken from stationary phase cultures of Sp7, and treated with DNase I as described in RNA extraction and RT-PCR section. The transcripts of both genes were reverse transcribed into cDNA using *gca1 *gene-specific primer, gcaR1 (Table 1, and Figure 4C), as *argC *and *gca1 *were predicted to be co-transcribed. The cDNA was purified using High Pure PCR product purification kit (Roche) and poly (dA) tailed at their 3' ends. The resulting poly(dA)-tailed cDNA was used as template in two different PCR reactions designed to amplify 5' end of *gca1 *and *argC *using oligodT-anchor/gcaR2 and oligodT-anchor/argR1 primer sets, respectively. The oligo dT-anchor primer was provided by the kit to anneal at the poly(dA) tail and gcaR2 (Table 1, and Figure 4C) was complementary to a region upstream of the gcaR1 binding site. The products of the first PCRs were separately used as template in second PCRs using anchor/gcaR3 and anchor/argR2 primer sets. Anchor primer was provided by the kit to anneal at a region generated by oligo dT-anchor primer at 3' end of cDNA, and gcaR3 and argR2 (Table 1, and Figure 5C) were further complementary to the region upstream of the gcaR2 and argR1 binding sites, respectively. The amplified product obtained was ligated into the pGEM-T Easy vector (Promega) and the nucleotide sequence of several distinct clones was determined in an ABI-PRISM™, 310 Genetic Analyzer (Applied Biosystems) using T7 forward and Sp6 reverse universal primers.

### Construction of promoter: *lacZ *fusions

Chromosomal region of *A. brasilense *(- 455 to + 79 of TSS) encompassing TSS and promoter elements for *argC *was PCR amplified using argPrF/argPrR primers (Table 1), and inserted between *Kpn*I and *Stu*I site of pRKK200 to construct a promoter:*lacZ *fusion (transcriptional fusion). In order to examine if *gca1 *has its own separate promoter, the upstream region from -501 to -11 of the predicted translational start site of *gca1 *was amplified using gca1PrF/gca1PrR primers and cloned in pRKK200 in a similar way. In both cases amplified products were digested with *Kpn*I/*Stu*I, and ligated with similarly digested pRKK200 vector. *E. coli *DH5α was then transformed with the ligation mix and the transformants were selected on Luria agar supplemented with kanamycin (100 μg/ml). After confirmation of recombinant plasmids by sequencing, the constructs were designated as pSK8 (P*_argC_***:***lac*Z fusion) and pSK9 (P*_gca1_***:***lac*Z fusion) (Table 2). These constructs were finally conjugatively mobilized into *A. brasilense *Sp7 via *E. coli *S.17.1 and exconjugants were selected on MMAB plates supplemented with kanamycin.

### β- Galactosidase assay

β-galactosidase assay [27] was performed with the cells of *A. brasilense *Sp7 harbouring either pRKK200, pSK8 or pSK9, and grown in MMAB under different conditions. To determine the effect of growth phase aliquots of cells were collected from exponential (0.7 to 0.9 OD_600_) and stationary phase (2.3 to 2.5 OD_600_). To examine the effect of CO_2 _concentration, above cells were grown in ambient air (0.035%) and high CO_2 _(3%) atmosphere. In order to study the effect of exogenous arginine, the cells were grown in MMAB supplemented with 0.1, 0.5 and 1 mM arginine. At the time of assay, the number of cells in each culture was equalized by diluting with either fresh medium or fresh medium supplemented with respective agents. The assay was performed with 1 ml of equalized culture in triplicate for each sample on two different occasions.

## Authors' contributions

SK did bioinformatic analysis, performed most of the experiments and drafted the manuscript. MNM designed the experiments, participated in performing RT-PCR and 5'RACE experiments and was involved in writing the manuscript. AKT conceptualized this study and supervised the experimental work, analysis of data, and preparation of the manuscript. All authors have read and approved the final manuscript.

## Supplementary Material

Additional file 1 **Comparison of the deduced amino acid sequence of γ-CA of *A. brasilense *(Gca1) with Cam, the prototypic γ-class CA from *M. thermophila***. The sequences were aligned using Clustal W. The conserved Zn ligands His-81, His-117 and His-122 are indicated in dark shaded boxes. Arg-59, Asp-61 and Gln-75, shown in light shaded boxes, are completely conserved residues in all γ-CA sequences. Numbers indicating residue positions refer to the position in the *M. thermophila *sequence lacking signal sequence Click here for file
