# Supplementary material for: Gene encoding γ-carbonic anhydrase is cotranscribed with argC and induced in response to stationary phase and high CO2 in Azospirillum brasilense Sp7
Source: BMC Microbiol. 2010 Jul 4;10:184. doi: 10.1186/1471-2180-10-184 (PMC2914000; doi:10.1186/1471-2180-10-184)
Supplement: Additional file 1 — Comparison of the deduced amino acid sequence of γ-CA of A. brasilense (Gca1) with Cam, the prototypic γ-class CA from M. thermophila. The sequences were aligned using Clustal W. The conserved Zn ligands His-81, His-117 and His-122 are indicated in dark shaded boxes. Arg-59, Asp-61 and Gln-75, shown in light shaded boxes, are completely conserved residues in all γ-CA sequences. Numbers indicating residue positions refer to the position in the M. thermophila sequence lacking signal sequence [file 1471-2180-10-184-S1.PDF]

```
                                                                          59
Gca1            -----------MSGLILPFQG--THPKIDPSVYVAPTASVIGDVEIGPGSSVWFGCTIRG 47
Cam             QEITVDEFSNIRENPVTPWNPEPSAPVIDPTAYIDPQASVIGEVTIGANVMVSPMASIRS 60
                            .. : *::   : * ***:.*: * *****:* **..  *   .:**.

                61/62         75    81                                   117
Gca1            D-VNEIRIGARTNIQDGTVIHVASAGQG------------------TYIGDDVSIGHMAL 88
Cam             DEGMPIFVGDRSNVQDGVVLHALETINEEGEPIEDNIVEVDGKEYAVYIGNNVSLAHQSQ 120
                *    * :* *:*:***.*:*. .: :                   .***::**:.* :

                 122
Gca1            LH-ACTLEGGCFIGMQACVMD----------------GAYVESGAMVAAGALVT------ 125
Cam             VHGPAAVGDDTFIGMQAFVFKSKVGNNCVLEPRSAAIGVTIPDGRYIPAGMVVTSQAEAD 180
                :* ..:: .. ****** *:.                *. : .*  :.** :**

Gca1            -------------------------------PGKRVATGQLWAGSPARPVRALTEKDTSF 154
Cam             KLPEVTDDYAYSHTNEAVVYVNVHLAEGYKETSKDVPPRSLVMGVPAKIIKQVSEGQVQG 240
                                               ..* *.. .*  * **: :: ::* :..

Gca1            FPVNIRNYVRLAQIYREG--- 172
Cam             LLDHGQNYVRLAKAHAAAGLG 261
                :  : :******: :  .
```
